# Clinical and histological features of gingival lesions: A 17-year retrospective analysis in a northern Italian population

**DOI:** 10.4317/medoral.17809

**Published:** 2012-02-09

**Authors:** Mario Carbone, Roberto Broccoletti, Alessio Gambino, Marco Carrozzo, Carlotta Tanteri, Pier L. Calogiuri, Davide Conrotto, Sergio Gandolfo, Monica Pentenero, Paolo G. Arduino

**Affiliations:** 1 Department of Biomedical Sciences and Human Oncology, Oral Medicine Section, Lingotto Dental School, University of Turin, Turin, Italy; 2 Department of Oral Medicine, School of Dental Sciences, University of Newcastle upon Tyne, Newcastle upon Tyne, UK; 3 Department of Clinical and Biological Sciences, Oral Medicine and Oral Oncology Section, University of Turin, Orbassano, Italy

## Abstract

Objectives: Only few studies on gingival lesions considered large enough populations and contemporary literature does not provide a valid report regarding the epidemiology of gingival lesions within the Italian population. The histopathological and clinical appearance of 538 gingival lesions from northern Italians are described and discussed here. Study Design: The case records of patients referred for the diagnosis and management of gingival lesions, from October 1993 to October 2009, were reviewed. Data regarding the histological type of lesion were also obtained from the biopsy register for each case, and blindly re-examined. Results: We reported a greater frequency of benign lesions (reactive and/or inflammatory) in non-plaque/non-calculus induced gingival disorders. We confirmed an unambiguous prevalence of oral squamous cell carcinoma above all other malignant neoplasia, and a prevalence of neoplastic malignant lesions in the maxilla, with a slight increase in females and a drift of the incidence peak from the seventh to the eighth decade. There was a prevalence of precancerous gingival lesions in the maxilla, with a higher incidence in females and with a drift from the sixth to the seventh decade. We also reported a prevalence of oral lichen planus and lichenoid lesions as major manifestations of desquamative gingivitis. Conclusions: The high frequency of gingival involvement of such different diseases emphasizes the importance of histological characterization and differential diagnosis for periodontists, but more prospective studies are needed to better describe the true incidence of the non-plaque related gingival diseases.

** Key words:**Gingival lesions, clinical appearance, histological analysis.

## Introduction

The gingiva is commonly affected by non-neoplastic and neoplastic lesions, the latter usually being characterized by a progressive growth that can be either benign or malignant. Non-neoplastic lesions are either inflammatory or represent a reaction to diverse types of irritative stimuli. Moreover, the great majority of localised overgrowths of the gingival are considered to be reactive rather than neoplastic in nature (1-3). Desquamative gingivitis manifests predominantly as painful erosions or ulceration of the attached and free gingiva. It is now known to represent a common manifestation in a number of disorders ranging from bullous diseases to adverse reactions to a variety of chemicals or allergens (4). An accurate comparison and evaluation of the findings obtained by previous epidemiological studies is difficult because of the limited number of reported cases; this is the reason why estimating the prevalence of gingival lesions worldwide proves to be an arduous task. Up to this date, few studies on gingiva lesions considered large enough populations and contemporary literature does not provide a valid report regarding the epidemiology of gingiva lesions within the Italian population. The aim of this study was that of analysing clinical and histological features of gingival lesions in a Northern Italian population and to compare obtained data with those previously collected.

## Material and Methods

The case records of all patients, who had been initially referred to the Oral Medicine Unit of the main hospital of the city of Turin, Italy, for the diagnosis and management of gingiva lesions, from October 1993 to October 2009, were reviewed and relevant retrospective data selected and extracted. Demographic information, age and gender, smoking, alcohol consumption, clinical aspect of the lesions, and sites of oral involvement were collected. To complete the final list, the following inclusion criteria were adopted: 1) all age groups and both sexes; 2) reports with complete and satisfactory case histories; 3) tissue samples obtained exclusively from gingiva, edentulous fibromucosa or alveolar mucosa; 4) more than one sample for a given patient, as long as biopsied at different times.

Data regarding the histological type of lesion were obtained from the biopsy register for each case. Haematoxylin and eosin sections of each specimen were evaluated by light microscopy and blindly reexamined by an expert oral pathologist.

The diseases were then classified into 4 groups by an expert oral physician (M.C.): a] neoplastic lesions (both malignant and benign); b] non-neoplastic lesions; c] oral pre malignant lesions; d] lesions caused by autoimmune diseases.

All analyses were performed using SPSS® software (SPSS for windows, version 11, SPSS inc, Chicago, IL, USA). All different data collected from each patient were later analyses using descriptive statistics. Continuous variables are expressed as mean ± SD.

## Results

The study group involved 448 non-related Caucasian patients (269 females and 179 males) and 1 Afro-Caribbean female (F : M = 1.5 : 1). The mean age at presentation was 57.5 years for men (SD ± 13.6) and 54.1 years for women (SD ± 12.8). The total number of biopsies samples resulted to be 538 and records showed that 68 patients underwent biopsy more than once ( Table 1).

a. Neoplastic lesions ( Table 2).

**Table 1:**
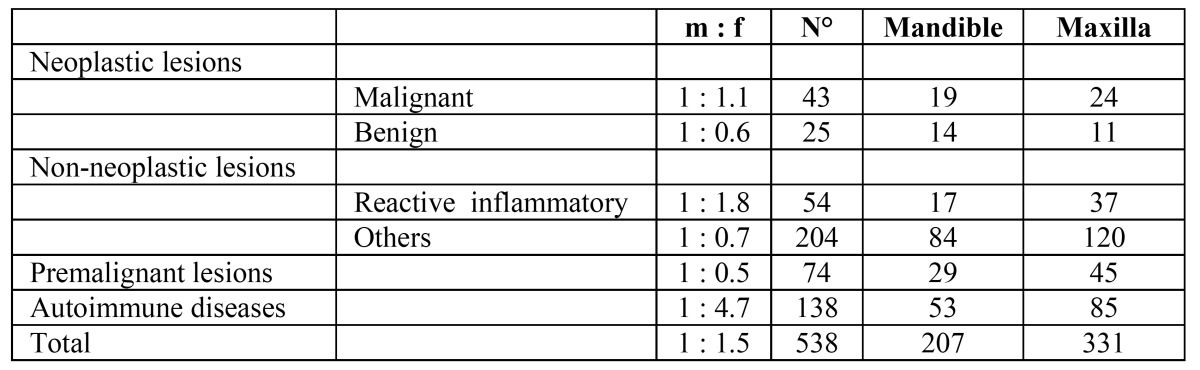
Frequency and site distribution of 538 gingival specimens.

**Table 2:**
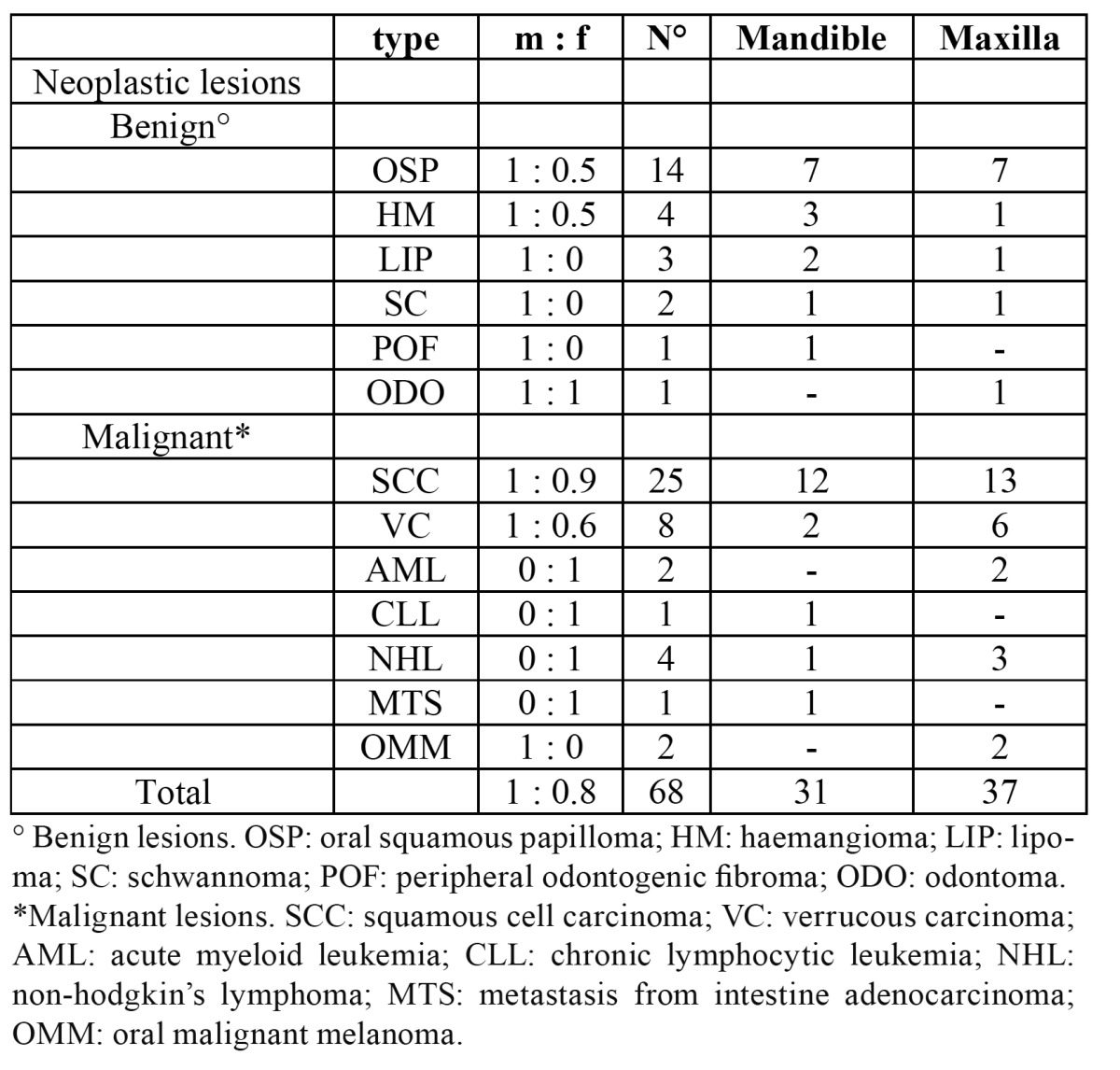
Frequency and site distribution of neoplastic gingival lesions.

**Figure 1:**
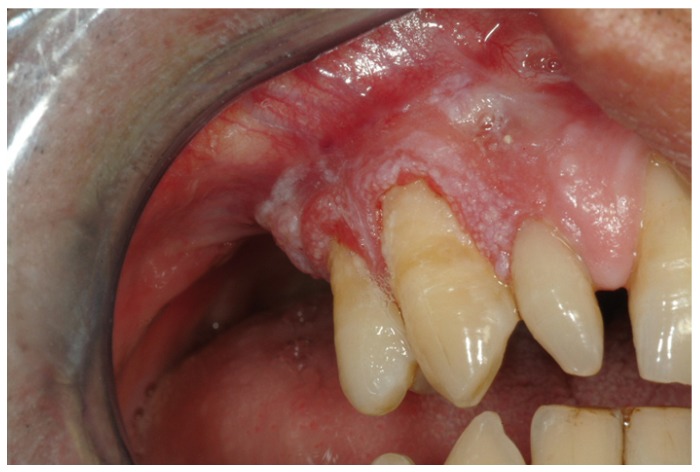
Clinical view of a squamous cell carcinoma on the right maxillary premolar gingival area.

**Figure 2:**
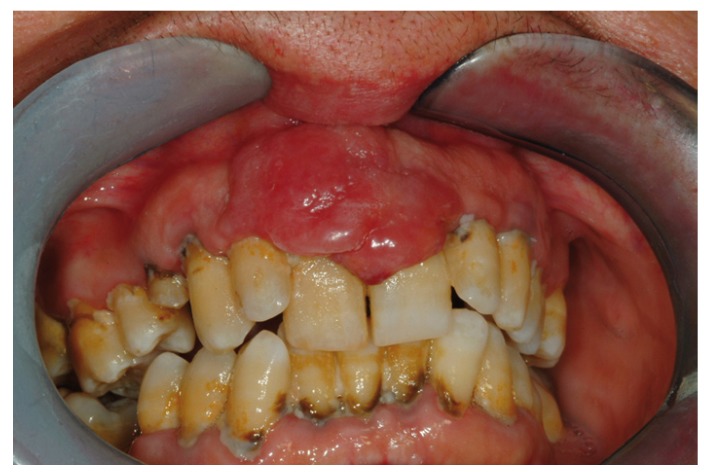
Clinical view of an upper gingival case of B cell non-Hodgkin lymphomas.

**Figure 3:**
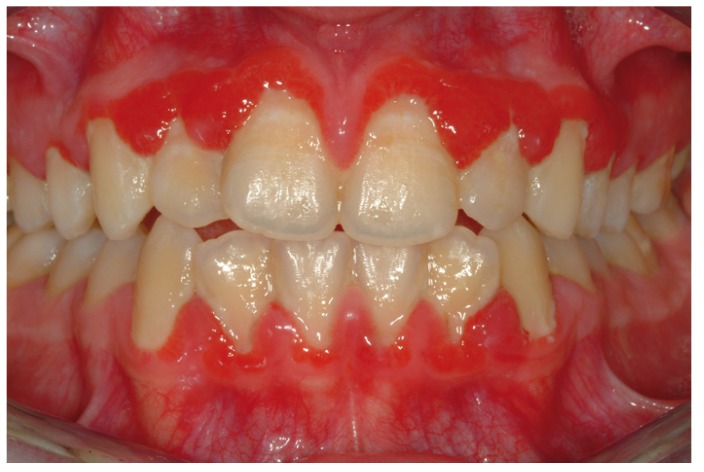
Clinical view of a diffuse gingival involvement in a patients with a diagnosis of plasma cell gingivitis.

Malignant neoplastic lesions accounted for almost 8% of all biopsied gingiva lesions. Twenty-five out of 43 malignant tumours were diagnosed as oral squamous cell carcinoma (60%) (Fig. 1), 8 resulted to be a verrucous carcinoma (19%), 4 were B cell non-Hodgkin lymphomas (Fig. 2, 3) ,2 were diagnosed as acute myeloid leukaemia, 2 as melanoma, 1 as chronic lymphocitic leukaemia and 1 resulted to be a metastasis of a primary intestinal adenocarcinoma. When considering sampling sites, the majority of surgical samples were obtained from the maxillary attached gingiva (n=17), followed by the mandibular alveolar mucosa (n=13), the maxillary alveolar mucosa (n=7) and finally the mandibular attached gingiva (n=6). When considering the 25 cases of squamous cell carcinoma, the male to female ratio resulted to be nearly 1:1, whereas in verrucous carcinoma it was equal to 1.7:1. Leukaemia, lymphoma and metastases were found in females only; on the other hand, melanomas were diagnosed exclusively in men. The highest number of malignant lesions was detected in patients aged 60 to 69 whilst no manifestation of such lesions was found in patients under the age of 20. Benign neoplastic lesions were classified as squamous papilloma (56%), hemangioma (16%), lipoma (12%), schwannoma (8%), peripheral odontogenic fibroma (4%), odontoma (4%). The most commonly involved localization resulted to be the maxillary attached gingiva, with a higher incidence in males and two incidence peaks (fifth and eight decade).

b. Non-neoplastic lesions ( Table 3).

**Table 3:**
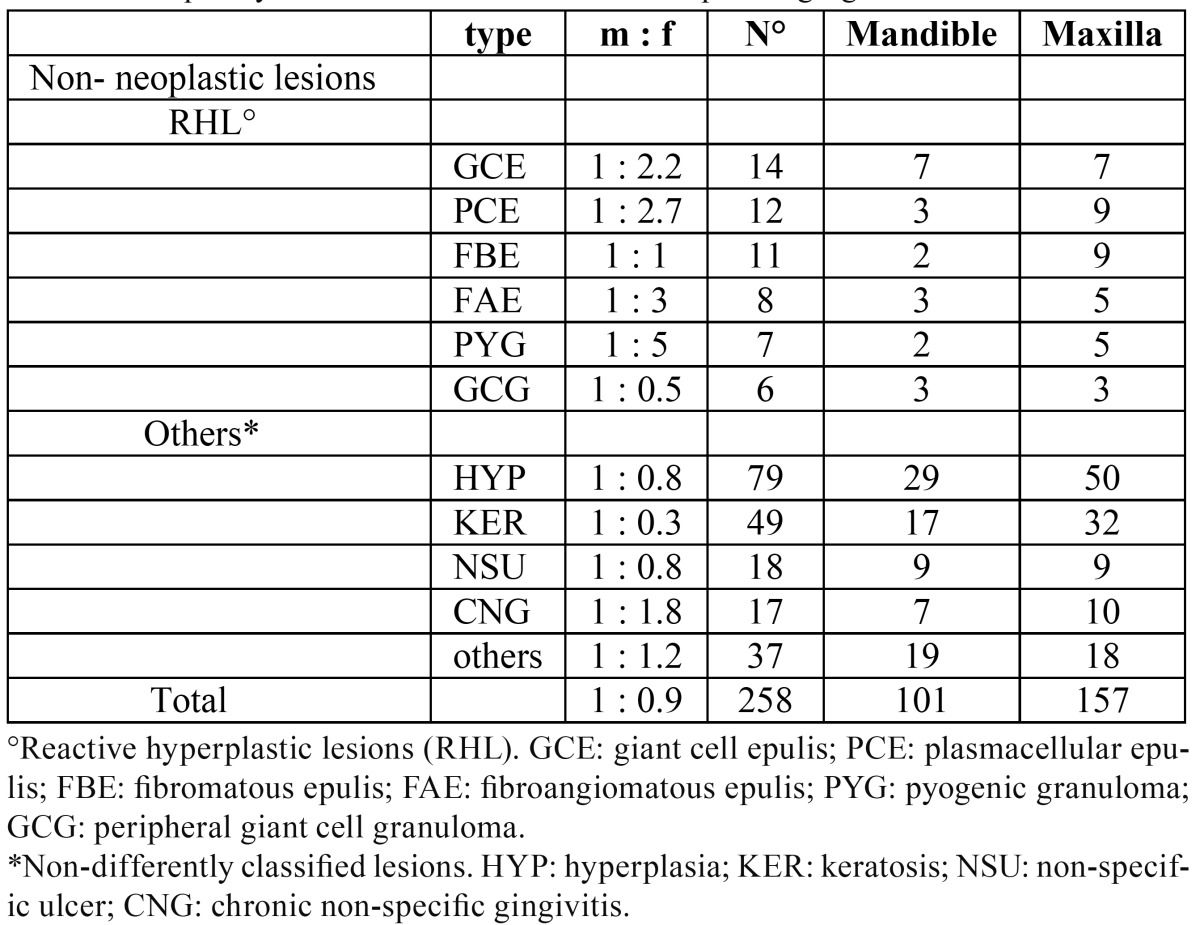
Frequency and site distribution of non-neoplastic gingival lesions.

Mainly localized reactive hyperplastic lesions belong to this group. They were classified as follows: giant cell epulis (24%), plasmacellular epulis (20%), fibromatous epulis (15%), fibroangioma epulis (15%), pyogenic granuloma (11%), peripheral giant cell granuloma (11%). The maxillary attached gingiva appeared to be the most commonly involved site and females represented the majority of patients, with an incidence peak in the fourth decade. Amongst the other encountered pathologies we diagnosed undifferentiated hyperplasia (38%), keratosis (23%), nonspecific ulcer (9%), chronic nonspecific gingivitis (9%), and a smaller number of periodontal cysts, amalgam tattoos, melanotic maculae, racial pigmentation, lentigo simplex, sarcoidosis and amyloidosis (all ranging between 3 and 1%). Such lesions were mostly seen in males with the exception of cysts and chronic gingivitis. They reached a peak of incidence around the seventh decade and involved males and females almost equally (47% and 53% respectively).

c. Oral pre malignant lesions

Pre malignancies represented almost 14% of all lesions (74 overall). The following encountered lesions belong to this group: leukoplakia without dysplasia 86.4% (n=64), leukoplakia with mild dysplasia (n=7), leukoplakia with moderate dysplasia (n=2), and erythroplakia with severe dysplasia (n=2). The elective localization of such lesions appeared to be the attached gingiva of the maxilla (51.3%). The majority of subjects to undergo a biopsy and to obtain the subsequent diagnosis of pre cancerous lesion were females (67.8%). When considering age distribution, pre malignant lesions showed an incidence peak around the seventh decade, even though these lesions can be detected at any age. Conversely, dysplasia reached an incidence peak around the eight decade.

d. Autoimmune diseases ( Table 4).

**Table 4:**
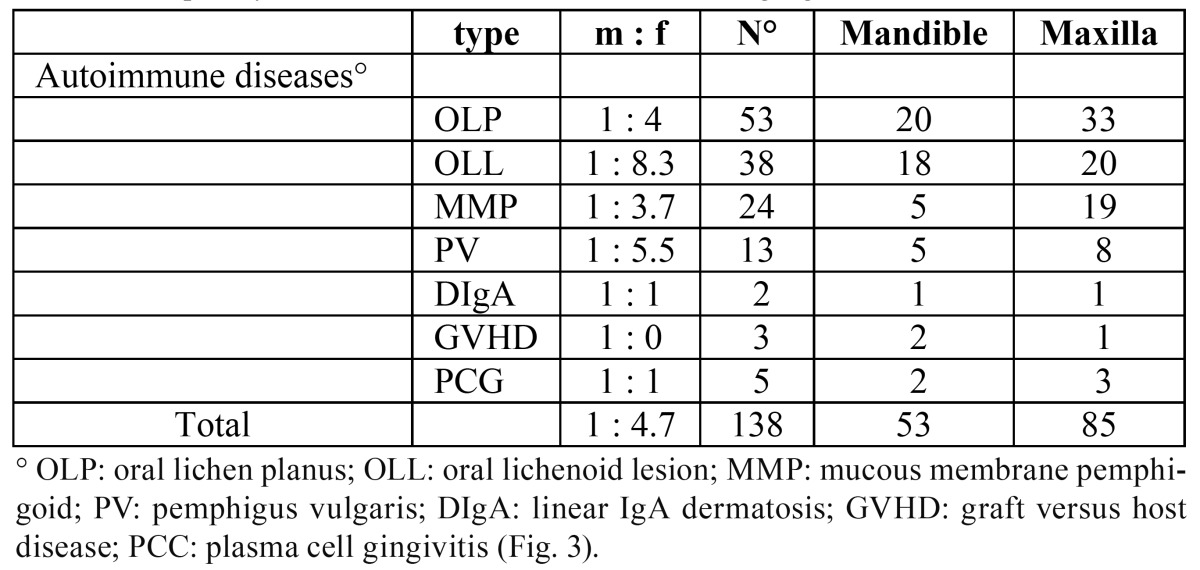
Frequency and site distribution of autoimmune gingival diseases.

Oral lichen planus showed to be the most frequent pathology, representing 38.4% of all disimmune diagnoses, followed by oral lichenoid lesions (27.5%), mucous membrane pemphigoid (17.4%) and pemphigus vulgaris (9.4%). The attached gingival was the most commonly involved tissues (64%); an exception to this was represented by pemphigoid, as the majority of tissue samples were taken from the maxillary alveolar mucosa. Females were the most represented group (81.1%) and the incidence resulted to be higher in the sixth decade.

## Discussion

To the best of our knowledge, this study represents the first report on the frequency and distribution of biopsied gingiva lesions within the Northern Italian population. The clinical and histological features of patients in our survey share many similarities with those reported previously in other countries, but we also reported some essential differences.

The majority of samples (37.2%) led to the diagnosis of non-neoplastic lesions (inflammatory reactive lesions and other non-definable nor specific lesions). Within this group, gingiva hyperplasia resulted to be the most common histopathological manifestation. Such results agree with those previously reported in the literature (5-9). Although the total number of those gingiva lesions was higher in females, non-neoplastic lesions were equally distributed among males and females, with an insignificant prevalence in the former group. Moreover, our results have pointed out an incidence peak, concerning all non-neoplastic lesions, in patients aged 60 to 69. In contrast with this, two analogous studies had previously highlighted how the majority of biopsies had been carried out in patients aged 30-39 (8), and 20-29 (5). The difference between these results and ours is probably bound to the different criteria adopted to classify lesions. The present study does, in fact, subdivide non-neoplastic lesions into reactive hyper plastic and non-definable/specific lesions, whereas other authors merge all non-neoplastic benign lesions into one group. Interestingly enough, when considering our results concerning reactive inflammatory lesions alone, we have noticed an incidence peak between 30 and 39 years of age, thus consistently in agreement with the previously mentioned studies.

Malignant neoplastic lesions represented 8% of all diagnosed gingival lesions. Such a finding is different from the incidence reported in the literature, ranging from 1 to 3.5% (5,8-19). Amongst these, Layfield and co-workers in particular carried out more than 30.000 gingival biopsies over a period of 24 years and reported an incidence of malignant lesions equal to 1.4% (8). Such a noteworthy difference certainly underlines how all those authors tend to include periodontal disorders into the considered gingiva lesions, thus occupying the majority of the sample. When considering the distribution of disorders within the malignant neoplasia group, we found squamous cell carcinoma to prevail on all the others, similarly to previous findings (6,8,12-19). Data analysis revealed that the second most common diagnosis of malignancy resulted to be that of verrucous carcinoma with an incidence of 19% out of all malignant neoplasias, according to our previous published data (20,21). Although the literature generally describes the attached gingiva and the alveolar mucosa in the mandible as being the preferential location of malignant neoplasia, and the mandible for all different types of oral tumour (12,13), the data collected in the present study actually highlighted the prevalence of such type of malignant lesions in the attached gingiva and alveolar mucosa of the maxilla. The analysis of our archives did, in fact, reveal 26 biopsies in the maxilla and 17 in the mandible that finally led to such diagnosis. The latter would appear to be the most original and significant result of our work, and it certainly is in contrast with previously published studies, even though we cannot supply any clear nor univocal interpretation about it. Oral melanoma has previously been reported with a frequency varying from 0.2 to 8% (22) but more recent data are missing; within the population considered by this study, its frequency was equal to 5%. Oral malignant melanomas mostly occur in the palate and gingiva, with the maxillary arch affected in most of the cases, and our results confirmed this. When considering data regarding the distribution of malignancies according to age and gender, our results do reflect those reported in the literature. All, in fact, agree on emphasizing a slight and progressive increase of malignancies in females over the last decade (12,18). Such an event is probably owed to the fact that they are now more exposed to risk factors than they were in the past. In addition to this, the incidence peak has moved from the seventh to the eight decade of human life as our race is surviving for longer (18).

In our report, pre cancerous lesions represented almost 14% of all biopsies; 14.8% of these were diagnosed as epithelial dysplasia of different degrees and such a result is in agreement with Layfield and coworkers (8). Previous studies described the prevalence of leukoplakia without dysplasia to range between 18 and 38% (23-26), whilst the present study has detected a prevalence of gingival leukoplakia of 86.4% within the considered population. Such a result is justified by a complete lack in the literature of an analysis on leukoplakias localized solely on the gingiva. In fact, all the above-mentioned studies took care of evaluating the distribution of leukoplakia within the whole of the oral cavity. In this case gingiva and alveolar mucosa occupied the third and fourth place respectively, following floor of the oral cavity, tongue and vestibular mucosa. These studies do, however, describe a slight prevalence in the mandible and this is once again in contrast with our results.

The existing classification system for periodontal diseases includes, among non plaque–induced gingival disorders, ‘‘gingiva manifestations of systemic conditions’’. Several mucocutaneous disorders (e.g., oral lichen planus, mucous membrane pemphigoid, pemphigus vulgaris, erythema multiforme, lupus erythematosus and others) are listed in this subgroup together with allergic reactions (27). Besides their heterogeneous nature, all of these diseases share two common characteristics: an immunomediated pathogenesis and a similar clinical appearance with erythematous lesions, blisters, erosions, and ulcers, mainly located on the attached gingiva and palatal mucosa. Those clinical features are described as “desquamative gingivitis” (DG), one of the most important manifestations of disimmune diseases. The attached gingiva and the alveolar mucosa are preferential sites for biopsy in all DGs. Clinical features of desquamative gingivitis in relation to the most common disimmune disorders have been highlighted by two studies in particular (28). These have shown that the most frequently associated disease is pemphigoid, followed by oral lichen planus and by pemphigus vulgaris. Within the population included in the present study, desquamative gingivitis was most frequently associated with oral lichen planus and lichenoid lesions, followed by mucous membrane pemphigoid and pemphigus vulgaris. The elevated number of cases presented by our study makes such a result original. We have observed a prevalence of disimmune disorders in females and an incidence peak in the age range 60-69. Whilst the former result is in agreement with the literature (29), the latter is in contrast with the study conducted by Lozada and coworkers (30), who reported a higher frequency in females aged 50-59. None of the previously published researches clearly described the localisation and distribution of gingival manifestations of disimmune disorders. The present study does, instead, underline a neat prevalence on the attached gingiva and alveolar mucosa of the maxilla. Such a result could be bound to a technical choice carried out by the operator: it is possibly easier and simpler to carry out a maxillary rather than a mandibular biopsy of the soft tissues (the majority of such disorders has, in fact, multiple localisations).

In conclusion, our results confirm a greater frequency of benign lesions in non-plaque/non-calculus induced gingival disorders. We confirmed an unambiguous prevalence of oral squamous cell carcinoma above all other malignant neoplasia, and, for the first time ever, an absolute prevalence of neoplastic malign gingival lesions in the maxilla. We do confirm the slight increase of incidence of oral malignancies in females and a drift of the incidence peak from the seventh to the eighth decade. This research is the first to highlight a prevalence of pre cancerous gingival lesions in the maxilla, which takes place with a higher incidence in females and with a drift from the sixth to the seventh decade. Finally, this study is original and unique in showing a prevalence of oral lichen planus and lichenoid lesions as major manifestations of desquamative gingivitis in a cohort of patients from the same clinic. The high frequency of gingival involvement of such different diseases emphasizes the importance of histological characterization and differential diagnosis for periodontists, but more prospective studies are needed to better describe the real incidence of non-plaque related gingival diseases.
